# The role of *ABCA7* in Alzheimer’s disease: evidence from genomics, transcriptomics and methylomics

**DOI:** 10.1007/s00401-019-01994-1

**Published:** 2019-03-22

**Authors:** Arne De Roeck, Christine Van Broeckhoven, Kristel Sleegers

**Affiliations:** 1 Neurodegenerative Brain Diseases Group, VIB Center for Molecular Neurology, University of Antwerp, CDE, Universiteitsplein 1, 2610 Antwerp, Belgium; 2 Biomedical Sciences, University of Antwerp, Antwerp, Belgium

**Keywords:** ATP-binding cassette, sub-family A, member 7 (ABCA7), Alzheimer’s disease, Meta-analysis, Loss-of-function, Endophenotype, Rare variants, Variable number tandem repeat (VNTR)

## Abstract

Genome-wide association studies (GWAS) originally identified ATP-binding cassette, sub-family A, member 7 (*ABCA7*), as a novel risk gene of Alzheimer’s disease (AD). Since then, accumulating evidence from in vitro, in vivo, and human-based studies has corroborated and extended this association, promoting *ABCA7* as one of the most important risk genes of both early-onset and late-onset AD, harboring both common and rare risk variants with relatively large effect on AD risk. Within this review, we provide a comprehensive assessment of the literature on *ABCA7*, with a focus on AD-related human -omics studies (e.g. genomics, transcriptomics, and methylomics). In European and African American populations, indirect *ABCA7* GWAS associations are explained by expansion of an *ABCA7* variable number tandem repeat (VNTR), and a common premature termination codon (PTC) variant, respectively. Rare *ABCA7* PTC variants are strongly enriched in AD patients, and some of these have displayed inheritance patterns resembling autosomal dominant AD. In addition, rare missense variants are more frequent in AD patients than healthy controls, whereas a common *ABCA7* missense variant may protect from disease. Methylation at several CpG sites in the *ABCA7* locus is significantly associated with AD. Furthermore, *ABCA7* contains many different isoforms and *ABCA7* splicing has been shown to associate with AD. Besides associations with disease status, these genetic and epigenetic *ABCA7* markers also showed significant correlations with AD endophenotypes; in particular amyloid deposition and brain morphology. In conclusion, human-based –omics studies provide converging evidence of (partial) ABCA7 loss as an AD pathomechanism, and future studies should make clear if interventions on *ABCA7* expression can serve as a valuable therapeutic target for AD.

## Introduction

Dementia—and in particular Alzheimer’s disease (AD), which makes up 50–70% of dementia patients—is one of the major medical challenges of our time. There is a dire need for drugs to prevent, delay the onset, slow the progression, or treat the (symptoms of) disease. So far, AD drug development has been unusually difficult. Most efforts have focused on interventions in the amyloid cascade, the main AD pathology hypothesis, which postulates that amyloid β (Aβ) accumulation leads to synaptic deficits and tau aggregation which in turn causes neuronal death. While early tau pathology in the entorhinal and limbic system can manifest without any Aβ, severe neocortical AD-like tau pathology in the form of neurofibrillary tangles and neuritic plaques is not seen in absence of Aβ, indicating that Aβ is crucial for the development of clinically overt dementia [1]. Among other brain regions, pathological hallmarks often originate in the hippocampus, a brain region involved in the generation of memories. As a consequence, short-term memory loss is often one of the first and principal symptoms of AD [2]. Nevertheless, there is increasing awareness that current attempts to intervene in the amyloid cascade are insufficient. To overcome these issues, several efforts are needed, which include, but are not limited to: new therapeutic targets and better clinical trial design through development of biomarkers with high predictive value in the pre-dementia stage, readily accessible biomarkers, and better markers for reliable and timely diagnosis [1].

Generally, when studying the molecular biology of AD, it is not trivial to differentiate the disease-causing pathways from complementary processes. Identification of a gene with AD-associated variants, however, provides certainty about an early pathological role of that gene which forms a potentially interesting target for drugs. Additionally, the variants themselves are easily assessed using peripheral tissue (e.g. blood) and provide early markers which can be useful for prognosis and diagnosis. AD is split into two categories based on the disease onset age: early-onset AD (EOAD) and late-onset AD (LOAD), respectively, corresponding to patients with an onset age younger or older than 65 years of age. By studying rare autosomal dominant forms of EOAD, three genes (*APP*, *PSEN1,* and *PSEN2*) were found to harbor causal mutations. All of them are directly involved in the production of Aβ [3]. Most AD cases, however, are caused by a multifactorial etiology. *APOE*, a gene with pleiotropic effects, was identified as the first common genetic risk factor with high penetrance in EOAD and LOAD. Subsequently, genome-wide association studies (GWAS) have been conducted on large cohorts of unrelated LOAD patients and cognitively healthy controls. This led to the identification of common genetic variants with low AD-associated effect sizes in more than 20 loci. These common variants, however, only provide indirect association signals, and to understand the biology and clinical applicability, additional research is needed [4]. One of the loci with the highest post-GWAS research success rate is the ATP-binding cassette, sub-family A, member 7 (*ABCA7*) gene, with the identification of both common risk variants with a direct functional consequence on *ABCA7*, as well as rare coding variants of intermediate to high penetrance providing compelling evidence of the involvement of *ABCA7* in AD risk.

Further substantiation of the downstream molecular effects of *ABCA7* and the role of *ABCA7* in AD was initially obtained through in vitro and in vivo studies in model systems, and this has been the main topic of prior reviews on *ABCA7* [5–9]. While these in vitro and in vivo findings have been crucial to our understanding of *ABCA7*, they need to be translated to humans. No in vitro or in vivo model fully captures the entire AD spectrum, and in the past, this has led to a lack of concordance between animal models and human clinical trials [10]. While research based on human individuals has several practical limitations, they are paramount to understand the contribution and therapeutic potential for risk factors, such as *ABCA7*. Fortunately, many large-scale collaborations combining AD patient cohorts and biomaterials now exist. In combination with the development of high-throughput “omics” technologies (e.g. genomics, transcriptomics, and methylomics), this provides unprecedented insights into the molecular features of AD. Many of these novel findings trace back to *ABCA7*. In this review, we will first briefly discuss the main in vitro and in vivo findings, followed by a comprehensive assessment of human-based *ABCA7* studies.

### *ABCA7*, evidence from in vitro and in vivo models

**Fig. 1:**
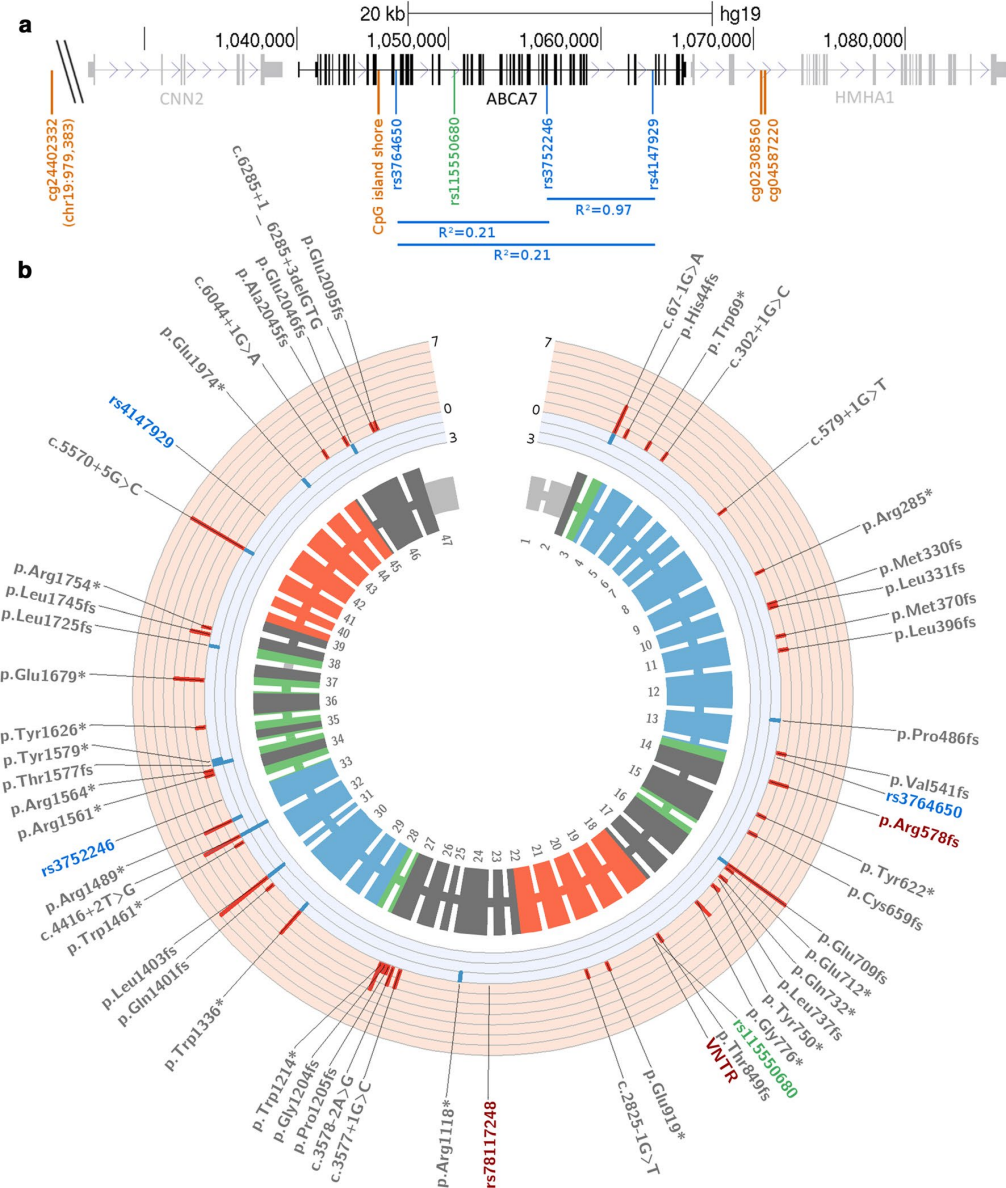
Genomic *ABCA7* layout. **a** Part of the *ABCA7* locus on chromosome 19 is shown (chr19:1026,000-1087,000 [hg19]), containing the *ABCA7* gene, flanked by the *CNN2* and *HMHA1* genes. Significantly, AD-associated CpG methylation markers (orange), and GWAS sentinel SNPs from African American (green) and Caucasian (blue) study populations are shown, with R^2^ LD values for the latter. **b** Detailed *ABCA7* plot, generated with circos [18]. From the outside to the inside: GWAS sentinel SNPs observed in African American (green), or Caucasian cohorts (blue), common functional variants (red), and PTC variants reported in AD case–control studies (gray) are shown. The subsequent track depicts the number of studies per PTC variant which report enrichment in AD (red, outward facing), or controls (blue, inward facing). The inner track corresponds to the 47 exons of *ABCA7* with protein annotation: UTR (light gray), transmembrane domains (green), extracellular domains (blue), NBD domains (red), and unknown protein domains (dark gray)

The *ABCA7* gene consists of 47 exons and encodes a complete transmembrane transporter protein of 2146 amino acids with a molecular weight of 220 kDa [11]. As a typical ABC transporter, *ABCA7* contains two intracellular nucleotide-binding domains (NBD) with conserved Walker A and B motifs for ATP hydrolysis, two transmembrane domains, and two extracellular loops (Fig. 1) [12]. A detailed ABCA7 structure (e.g. obtained through X-ray crystallography or cryogenic electron microscopy), however, is currently not available [RCSB PDB id: Q8IZY2, rcsb.org, accessed January 2019] [13]. Under physiological conditions, *ABCA7* is expressed in brain tissue at a generally low abundance, which is comparable to other human tissues and cell types [Genotype-Tissue Expression (GTEx) project, accessed January 2019]. Two studies examining *ABCA7* expression in mice brain, or human primary neurons from fetal brain, respectively, denoted the principal *ABCA7* expressing cell type as neurons [14] or microglia [15]. More recently though, with the advent of large-scale single-cell sequencing projects, *ABCA7* expression was observed to be relatively comparable across all different cell types in mouse brain [16]. A similar experimental set-up with human brain, unfortunately, did not detect sufficient *ABCA7* RNA levels to determine cell type-specific expression [17].

In analogy to ABCA1—the closest homolog to ABCA7 and a more extensively studied protein—ABCA7 is predicted to regulate lipid metabolism. In vitro studies show that both ABCA1 and ABCA7 conduct apolipoprotein A (apoA)-I mediated export of cholesterol and phospholipids, but with different preferred substrates and efficiencies [19–22]. Additionally, *ABCA7* expression is upregulated when cholesterol is depleted via the sterol regulatory element-binding protein 2 (SREBP2) pathway, which has an inverse regulatory effect on ABCA1 [23]. Furthermore, in vivo *Abca7* knockout mice have shown lower serum levels of cholesterol and high-density lipoprotein (HDL) [14], an altered phospholipid profile in the brain [24], and disruption of lipid rafts in thymocytes and antigen-presenting cells [25]. When analyzing *Abca7* deficiency in mouse primary macrophages, however, no effect on cholesterol and phospholipid efflux was observed [14]. Hence, while a role of ABCA7 in lipid metabolism seems very likely, more research is needed to understand the specific effects across different tissues and cell types, and in relation to pathological effects.

In addition to lipid metabolism, ABCA7 has been linked to the regulation of phagocytosis. This connection was hypothesized based on the homology between ABC transporters (in particular the ABCA sub-family) and *C. elegans* cell corpse engulfment gene *ced*-*7*, which is involved in the engulfment of cell corpses during programmed cell death [26]. While both ABCA1 and ABCA7 have similarity to CED-7, only depletion of *Abca7*, and not *Abca1*, affected phagocytosis in mouse macrophages in vitro and in vivo [27]. Upon *Abca7* deletion in mouse fibroblasts, phagocytosis was impaired [23]. Furthermore, *Abca7* knockout in AD mouse models showed increased amyloid deposition in the brain and reduction of oligomeric uptake of both Aβ_1-40_ and Aβ_1-42_ in macrophages and microglia [28, 29]. ABCA7 presumably affects phagocytosis of Aβ aggregates rather than soluble Aβ, since microdialysis studies showed no difference in clearance rate of soluble Aβ [24]. In regard to the expression of *ABCA7* across different cell types in the brain, as described above, it is important to note that in addition to microglia, also neurons, glial cells, and cerebrovascular cells are involved in Aβ uptake [30], and ABCA7 may play a role in them as well. Recently, Lamartinière et al. reported on a putative new role of ABCA7 as a regulator of cholesterol homeostasis and Aβ efflux at the blood–brain barrier [31]. Besides Aβ clearance, ABCA7 was also shown to directly affect Aβ production. Both in vitro and in vivo findings show an increase of Aβ upon suppression of ABCA7, presumably through β-secretase cleavage [32].

Finally, two in vivo studies assessed the role of *ABCA7* on cognitive and behavioral aspects of AD. Logge et al. examined *Abca7* knockout mice and observed impaired novel recognition memory in males and impaired spatial reference memory in females [33]. Impaired spatial memory was further corroborated in a cohort of male and female mice [24].

### Common genetic risk variants

In the last decade, several GWAS [34–40] were conducted on large cohorts of AD patients and controls, which introduced novel genetic risk loci for AD, including *ABCA7*. These studies provide AD-associated common single-nucleotide polymorphisms (SNPs), referred to as “sentinel” SNPs, which are presumed to be in linkage disequilibrium (LD) with a functional genetic variant. Because LD patterns and allele frequencies differ between populations, genetic association signals may differ between GWAS depending on the ethnicity of the cohorts. In Caucasians, three associated SNPs were pinpointed (Table 1, Fig. 1): intronic SNP rs3764650 (odds ratio (OR) = 1.2, Table 1) was the first sentinel SNP in the *ABCA7* locus to obtain genome-wide significance [35] and is the most frequently studied variant in *ABCA7* regarding the effect of common variants on (endophenotypes of) AD, as described below. A second Caucasian-based GWAS identified common missense variant rs3752246 (p.Gly1527Ala, OR = 1.2, Table 1) [38]. In silico analysis of rs3752246 suggests a benign effect (e.g. a Phred-scaled CADD score of 5.3, which ranks its deleteriousness at 30% of all genomic SNPs [41]). Finally, the largest peer-reviewed meta-analysis to date established intronic *ABCA7* SNP rs4147929 (OR = 1.1, Table 1) as AD-associated sentinel SNP. SNPs rs3752246 and rs4147929 are in complete linkage disequilibrium (LD) in European populations (*D*′ = 1.0, *R*^2^ = 0.97) [42]; hence, both tag the same functional variant. The LD between rs3764650 and rs4147929 (or rs3752246) is modest (*D*′ = 0.64, *R*^2^ = 0.21) [42], suggesting either variable haplotype structure of the same functional genetic variant between Caucasian populations, or tagging of distinct functional genetic variants. In African Americans, *ABCA7* intronic common variant rs115550680 was identified as AD-associated sentinel SNP. In contrast to Caucasian populations, rs115550680 (OR = 1.8, Table 1) had a much stronger effect size approaching that of *APOE* ε4 in African American populations [39]. Further research into the contribution of common *ABCA7* variants to AD in other ethnicities has mostly been limited to these sentinel SNPs. Meta-analysis of rs3764650 in a mixed population comprising five Asian cohorts and a Turkish cohort failed to show association [43]. These findings are most likely explained by different haplotype structures between these populations and/or the relatively small cohorts used in these studies (1871 patients and 3406 controls). Larger studies and hypothesis-free analysis of common variants are needed to understand the role of *ABCA7* in non-Caucasian and non-African American populations.

**Table 1:** AD-associated *ABCA7* (epi-) genetic variation

Variation	Interpretation	Origin	OR	MAF_gnomAD_ (%)
Common risk-increasing variants				
rs3764650	Intronic GWAS sentinel SNP, low predicted functional effect	CA	1.2 [1.1–1.3]	8.4
rs4147929	Intronic GWAS sentinel SNP, low predicted functional effect	CA	1.1 [1.1–1.2]	17.1
rs3752246	Missense GWAS sentinel SNP p.Gly1527Ala, predicted benign	CA	1.2 [1.1–1.2]	17.0
rs78117248	NGS-identified intronic candidate SNP, low predicted functional effect	CA	2.1 [1.3–3.3]	2.4
*ABCA7* VNTR expansions	Reduced *ABCA7* expression, loss of exon 19 encoding an ATP-binding domain	CA	4.5 [1.3–24.2]	0.8
rs115550680	Intronic GWAS sentinel SNP, low predicted functional effect	AA	1.8 [1.5–2.1]	6.2
rs142076058	PTC variant p.Arg578 fs, loss-of-function	AA	1.8 [1.4–2.4]	5.7
Rare variants				
PTC variants	Loss-of-function	CA	2.6 [1.3–5.5]*	1.1**
PTC variants	Loss-of-function	AA	1.4 [1.0–1.9]	7.3**
Missense variants	Unclear, misfolding and/or increased protein degradation?	CA	1.8 [1.3–2.4]*	/
Common protective variant				
rs72973581	Missense variant p.Gly215Ser, change into evolutionary conserved amino acid	CA	0.6 [0.4–0.95]	6.3
CpG methylation				
cg02308560	Hypermethylation in AD, Polycomb repression, effect on *ABCA7* unknown	CA	Est. = 7.7 (s.e. = 1.7)	/
cg24402332	Hypermethylation in AD, effect on *ABCA7* unknown	CA	Est. = 12.4 (s.e. = 3.4)	/
cg04587220	Hypermethylation in AD, Polycomb repression, effect on *ABCA7* unknown	CA	Est. = 4.7 (s.e. = 1.4)	/
CpG island shore (chr19:1045074–1045679)	Hypomethylation in AD, effect on *ABCA7* unknown	CA	log2FC = − 0.28	/

Variants and CpG methylation sites in *ABCA7* with reported association to AD are shown along with their interpreted functional effect, the origin of the study cohort, odds ratios (OR) with 95% confidence intervals, and origin-matched minor allele frequencies (MAF) in community-dwelling individuals from gnomAD

*CA* Caucasian, *AA* African American, *Est.* regression estimate, *s.e.* standard error, *log2FC* log2 fold change,

*Meta-analysis OR from this review

**Cumulative frequencies

### Effects on endophenotypes

Ensuing identification of common AD-associated *ABCA7* variants, several studies were performed to further understand the effects of these variants on biomarkers of AD, brain morphology, and symptomatic consequences (Table 2). Focusing on endophenotypes instead of clinical diagnosis results in more homogeneous phenotype definitions, which may lead to more accurate results. In comparison with the abovementioned large-scale GWAS, however, these follow-up endophenotype studies are conducted on smaller study populations at different stages of the disease (e.g. cognitively healthy elderly, individuals with mild cognitive impairment (MCI), AD patients, and post-mortem brain analyses), and results should be interpreted accordingly.

**Table 2:** Effects of AD-associated *ABCA7* (epi-)genetic markers on endophenotypes

Study	AD-associated marker	Reported significant effect of the risk allele		Study size			
				CON	MCI	AD	PCA
Amyloid and tau pathology							
Shulman et al. [44]	rs3764650	Increased neuritic plaque burden		229	178	297 + 21*	
Hughes et al. [45]	rs3752246	Increased amyloid beta deposition		178			
Yu et al. [100]	cg04587220	Methylation correlates with increased brain amyloidosis		293**		447	
Yu et al. [100]	cg04587220	Methylation correlates with higher tau tangle density		293**		447	
Apostolova et al. [46]	rs3752246	Increased brain amyloidosis		332	496	159	
Ma et al. [47]	rs3764650	Decreased CSF Aβ_1–42_ levels		104	306		
Brain morphology							
Ramirez et al. [48]	rs3764650	Cortical and hippocampal atrophy		50	98		
Roshchupkin et al. [49]	rs4147929	Voxel-based morphometry in the left postcentral gyrus		4071			
Stage et al. [50]	rs3752246	Decreased mean medial temporal lobe gray-matter density in dementia patients				294	
Sinha et al. [51]	rs115550680	Dissociation in entorhinal cortex (EC) resting-state functional connectivity		36			
Wachinger et al. [52]	rs4147929	Brain asymmetry in the hippocampus		434	636	171	
Clinical symptoms							
Karch et al. [53]	rs3764650	Later age at onset and shorter disease duration		39		73	
Engelman et al. [54]	rs3764650	Interaction with *APOE* on memory		1153			
Engelman et al. [54]	rs3752246	Interaction with *APOE* on memory		1153			
Carrasquillo et al. [55]	rs3764650	Nominal association with posterior cortical atrophy variant of Alzheimer’s disease		2523		54	81
Carrasquillo et al. [56]	rs3764650	Memory decline for subjects who eventually developed MCI/LOAD		2262			
Nettiksimmons et al. [57]	rs3764650	Cognitive decline in females		3267			
Schott et al. [58]	rs3764650	Associated with posterior cortical atrophy variant of Alzheimer’s disease					302
Andrews et al. [59]	rs3764650	Lower Immediate Recall Test and reduced rate of decline in Symbol Digit Modalities Test		1626			
Monsell et al. [60]	rs4147929	Increase of symptomatic AD compared to asymptomatic		68		521	
Sinha et al. [51]	rs115550680	Behavioral generalization		36			

Overview of studies reporting a significant association between AD endophenotypes and *ABCA7* sentinel SNPs (rs-numbers) or CpG methylation markers (cg-numbers)

*CON* cognitively healthy controls, *MCI* mild cognitive impairment, *AD* Alzheimer’s disease, *PCA* posterior cortical atrophy

*21 individuals had other forms of dementia than AD

**Unspecified how many of these were cognitively healthy or MCI

### ABCA7 common risk alleles increase amyloid deposition

One of the major efforts to study the role of AD-associated sentinel SNPs on amyloid pathology was conducted in the frame of the Alzheimer’s Disease Neuroimaging Initiative (ADNI). Within ADNI, a continuously growing number of cognitively normal individuals, individuals with MCI, and AD patients are recruited and subjected to ^18^F-florbetapir positron-emission tomography (PET), which measures amyloid deposition in the brain of living subjects. In the first two ADNI studies, no significant association between *ABCA7* GWAS SNPs and amyloid deposition was found [61, 9]. However, in the latest ADNI study comprising the largest cohort, the risk allele of *ABCA7* sentinel SNP rs3752246 was significantly associated with increased amyloid deposition. Specifically, of all 20 AD risk SNPs under study, the effect size of *ABCA7* was the highest after *APOE*. Further analysis revealed that the effect of *ABCA7* on amyloidosis was significant in the cognitively healthy and MCI individuals, but not in the AD group, suggesting an early effect of *ABCA7* [46]. These findings are in line with a Pittsburgh compound B PET study on cognitively normal individuals which reports association of rs3752246 and amyloid deposition [45]. In addition, post-mortem brain silver staining showed increased neuritic plaque formation for carriers of the rs3764650 risk allele [44]. Finally, one study found decreased amyloid β (Aβ) levels in CSF of non-demented Aβ positive subjects with an rs3764650 risk allele; no association was observed with tau CSF levels [47]. Hence, overall, carrying an *ABCA7* risk allele increases amyloid deposition in the brain (Table 2). Currently, no evidence is available for a role of *ABCA7* sentinel SNPs in (phosphorylated) tau homeostasis, although this was studied to a lesser extent than amyloid pathology. No studies have yet investigated molecular markers of other pathomechanisms through which *ABCA7* could be involved in AD (e.g. lipid metabolism and immune-related endophenotypes) in the brain, CSF, and/or plasma.

### Increased brain atrophy in carriers of common ABCA7 risk alleles

Brain atrophy, which can be measured by magnetic resonance imaging (MRI), correlates with neuronal loss and as such has value in diagnosis of AD and monitoring of disease progression [62]. The correlation between AD GWAS SNPs and MRI measurements has been studied in several studies across different brain regions. Within the frame of ImaGene, 4071 non-demented individuals were subjected to hypothesis-free whole brain MRI analysis, and the volumetric results were correlated to genetic AD risk loci. Of all 19 genetic AD risk loci under study (including *APOE*), the strongest association—albeit not passing conservative multiple-testing thresholds—was obtained for the *ABCA7* rs4147929 risk allele and decreased gray matter in the left postcentral gyrus [49]. This suggests an effect on brain morphology by *ABCA7* in the asymptomatic stages of AD, yet further research reaching statistical significance is required. Wachinger et al. used MRI to study asymmetry in four pre-selected brain regions (hippocampus, amygdala, putamen, and caudate) and found significant association between rs4147929 and hippocampal asymmetry, particularly within the AD subgroup [52]. Additionally, studies with relatively smaller study populations reported associations between *ABCA7* GWAS SNPs and decreased gray-matter density in dementia patients [50], increased cortical and hippocampal atrophy in controls and individuals with MCI [48], and dissociation of entorhinal cortex resting-state functional connectivity based on functional MRI in cognitively healthy elderly [51]. In summary, evidence is presently linking AD-associated *ABCA7* common variants to increased brain atrophy already at preclinical stages of AD (Table 2). Further research to understand the consequences on the entire brain, however, is warranted.

### ABCA7 common risk alleles and cognition

Evidence for association between *ABCA7* and cognitive decline has been inconsistent; which is in line with other genetic risk factors of AD apart from *APOE*. On the one hand, correlations between *ABCA7* SNPs and clinical symptoms have been observed, yet based on relatively small cohorts, or with associations not passing multiple-testing correction. These include: association with baseline cognitive performance and linear rate of change [59], increased likelihood of clinical symptoms in individuals with AD neuropathology [60], later age at onset and shorter disease duration [53], and behavioral generalization deficits [51]. On the other hand, large-scale studies found no, or complex associations between *ABCA7* sentinel SNPs and cognitive decline. For instance, the Three-City Dijon study, a longitudinal study comprising 4931 individuals older than 65 years of age found no association between *ABCA7* common variants and global cognition, verbal fluency, visual memory, information processing, or literacy [63]. A second longitudinal study with 3267 female and 3026 male community-dwelling individuals found association between rs3764650 and cognitive decline, but only in females [57]. Third, in a study of 1153 middle-aged adults enriched for parental history of AD, again no association was found between *ABCA7* sentinel SNPs and cognitive decline. However, a multiple-testing-corrected significant interaction was observed between *APOE* ε4 and both rs3764650 and rs3752246, which are not in strong LD. Strikingly, this interaction was consistent with a ‘flip-flop’ effect [64]: the effect on memory of *ABCA7* genotypes is in opposite directions, depending on the *APOE* genotype [54]. Fourth, Carrasquillo et al. reported a significant association between the risk allele of rs3764650 and increased rates of memory decline in individuals with a final diagnosis of MCI or AD [56]. Hence, while overall the effects of *ABCA7* GWAS SNPs on cognition seem to be minimal, these SNPs may alter cognition in subgroups stratified on gender, *APOE*-status, or disease progression (Table 2).

Two studies examined the effect of common risk loci of AD on the risk of developing posterior cortical atrophy (PCA), an atypical form AD, in which patients manifest with progressive decline in visuospatial, visuoperceptual, literacy, and praxis skills [65]. Both studies observed a significant association of the *ABCA7* rs3764650 risk allele and increased risk for PCA (Table 2) [55, 58]. This risk effect did not differ from the risk to develop typical AD [58]. Additionally, Logue et al. assessed the shared common variant etiology between AD and age-related macular degeneration (AMD), the most common form of severe blindness and vision loss among the elderly. Within AMD, they found a weak association for *ABCA7* AD sentinel SNP rs3752246, albeit with a different direction of effect. Stronger association with AMD was observed for another *ABCA7* SNP (rs3752228), which was not in LD with any of the *ABCA7* AD sentinel SNPs, hence suggesting a possible role of *ABCA7* in AMD, but most likely through a different mechanism than AD [66]. Finally, a recent study examined the effect of *ABCA7* sentinel SNP rs4147929 on vascular dementia, ischemic heart disease, ischemic cerebrovascular disease, and lipid levels in 104,258 individuals from the Danish general population, but did not observe an association with these endophenotypes [67].

### Downstream pathological mechanisms

GWAS associations were paramount in establishing *ABCA7* as a risk gene for AD. Yet to understand which function of *ABCA7* is compromised in AD, and to translate this into potential therapeutic targets, the downstream pathophysiological mechanisms should be elucidated. Sentinel SNP rs3752246 corresponds to a missense variant, but as described above, this amino acid change is not expected to affect the function of *ABCA7* based on in silico prediction. Instead, a potential functional mechanism of common variants in *ABCA7* could be the alteration of *ABCA7* expression. In particular, a decrease of *ABCA7* would be in line with the previously described AD-related effects observed in vitro and in vivo, as well as the effect of rare *ABCA7* variants, which will be discussed below. According to the Genotype-Tissue Expression (GTEx) project [accessed December 2018], the minor alleles of rs3752246 and rs4147929 reduce the expression of *ABCA7* in multiple tissues, including brain regions such as the cerebellum and the cerebral hemisphere. In addition, Vasquez et al. reported reduced *ABCA7* expression in the anterior cingulate brain region for carriers of the rs3764650 risk allele in both AD patients and controls [68]. Allen et al., on the other hand, found no significant effect of rs3764650 on the expression in temporal cortex and cerebellum; assessed in a larger cohort of autopsied AD brains [69]. Hence, at the molecular level these sentinel SNPs do not provide sufficient insight into how genetic variation in *ABCA7* contributes to AD. To resolve this issue, it is important to identify the underlying functional genetic variants, which are tagged by the sentinel SNPs. Furthermore, since these functional variants may not be in perfect LD with the sentinel SNPs, and this LD structure may differ between populations, it may be beneficial to use the functional variants rather than the indirect sentinel SNPs for future prognostic and diagnostic purposes.

### Underlying functional genetic variants

First, while unlikely since each GWAS SNP tags many other variants in LD, the sentinel SNPs themselves could exert a pathological effect. One study modelled the rs3764650 (intron 13) and rs3752246 (exon 33) risk alleles in vitro. They identified possible promoter-enhancing capabilities for the rs3764650 major non-risk allele, and the rs3752246 risk allele led to increases in secreted Aβ40 and Aβ42, and β-secretase activity [70]. Nevertheless, since this study is based on the overexpression of *ABCA7* and *APP* in human embryonic kidney cells (HEK293) or Chinese hamster ovary (CHO) cells, there is no guaranteed relevance of these findings in human brain tissue. In contrast to the functional sentinel SNP hypothesis, a study performed second-generation sequencing of the entire genomic *ABCA7* locus, including introns and regulatory regions, to examine all genetic variations across the locus for association with AD. This study, performed on a Caucasian cohort, identified rs78117248 as the common SNP with the strongest AD association, comprising a twofold risk-increasing effect (Table 1). Furthermore, this variant was in very strong LD (*D*′ > 0.91) with all three Caucasian GWAS SNPs (rs3764650, rs4147929, and rs3752246), and conditional regression analysis showed that the sentinel SNP associations could be explained by rs78117248 [71]. This association was further replicated in an independent non-Hispanic White cohort [72]. While the genetic evidence is supportive of a role for this SNP in AD susceptibility, it is located deep intronic, and inspection of the local genomic context suggests that substitution of a single nucleotide at this position will have a minimal effect on protein function and expression. This suggested that another non-genotyped variation in *ABCA7* in LD with this SNP would be the true functional variant.

Recent work points towards a repetitive DNA sequence in *ABCA7* that was poorly covered in sequencing studies [73]. In intron 18, a variable number of tandem repeats (VNTR) was identified with highly variable lengths ranging from 300 bp to more than 10 kb. Carrying a risk allele of rs3764650 and rs78117248 was significantly correlated with longer VNTR lengths. Alleles longer than 5.6 kb were defined as expanded VNTRs, which had a 4.5-fold enrichment in AD (Table 1). VNTR alleles shorter than this cutoff did not seem to differ in length distribution between patients and controls. More than 7% of the AD patient cohort was observed to carry an expanded *ABCA7* VNTR. Furthermore, based on *ABCA7* transcript analysis in Epstein–Barr virus transformed lymphoblastoid cell lines of patients and controls, increasing VNTR length was associated with decreasing *ABCA7* expression. In addition, alternative splicing of the VNTR-flanking exons (exon 18 and exon 19) was examined and increasing VNTR length was significantly correlated with an exon 19 skipping isoform, which results in loss of an ATP-binding domain of *ABCA7*. These *ABCA7* transcription and splicing effects are potentially regulated through respective binding of transcription factors and splice factors to the VNTR core motif [73]. VNTR length showed an inverse correlation with Aβ_1-42_ levels, corroborating the evidence from sentinel SNPs that common genetic variation in *ABCA7* has an effect on Aβ. No significant associations were found in relation to total tau and phosphorylated tau in CSF of AD patients [73]. Further research is needed to delineate this VNTR’s contribution to AD in other populations. This is not trivial, due to the difficult detection of expanded tandem repeats. Yet, recent technological advances such as long-read sequencing can overcome these difficulties as demonstrated for the *ABCA7* VNTR with the use of whole-genome long-read sequencing [74].

The strong *ABCA7* GWAS signal observed in African Americans can be explained by another biological variant. By sequencing carriers of sentinel SNP rs115550680, a 44-bp exonic deletion (rs142076058, p.Arg578 fs) was identified which causes a frameshift in the *ABCA7*-coding sequence resulting in the formation of a premature termination codon (PTC) [75]. While the functional consequences of this deletion remain to be explored, the mutant transcript was detectable in blood of carriers, and a resultant truncated protein would lack both NBD domains, as well as nine predicted transmembrane domains. Follow-up of p.Arg578 fs in an African American case–control cohort showed a 1.8-fold enrichment (Table 1) in AD patients, with 15.2% of the patients carrying the deletion compared to 9.7% in controls [75]. The deletion was observed in all affected individuals of a large early-onset AD sibship of Caribbean Hispanic descent. An independent African American case–control study, however, could not replicate the association, with a carrier frequency of 9.2% in patients and 7.4% in controls. Differences in cohort ancestry between studies could be a potential explanation for this discrepancy [76].

### Rare *ABCA7* variants

A discovery that firmly confirmed *ABCA7* as a risk gene for AD was the enrichment of rare variants in the gene in AD patients. Numerous independent studies reported this observation in 2015, including hypothesis-free studies such as a large-scale imputation study [77] and a small exome-array association study [78], as well as targeted gene resequencing studies [71, 79]. Particularly rare PTC variants, comprising frameshift variants, nonsense variants, and variants causing out-of-frame splicing, were highly enriched in AD [80, 81, 75, 71, 82, 83, 72, 76, 84–86, 77, 79].

### Strong enrichment of PTC variants in AD

**Fig. 2:**
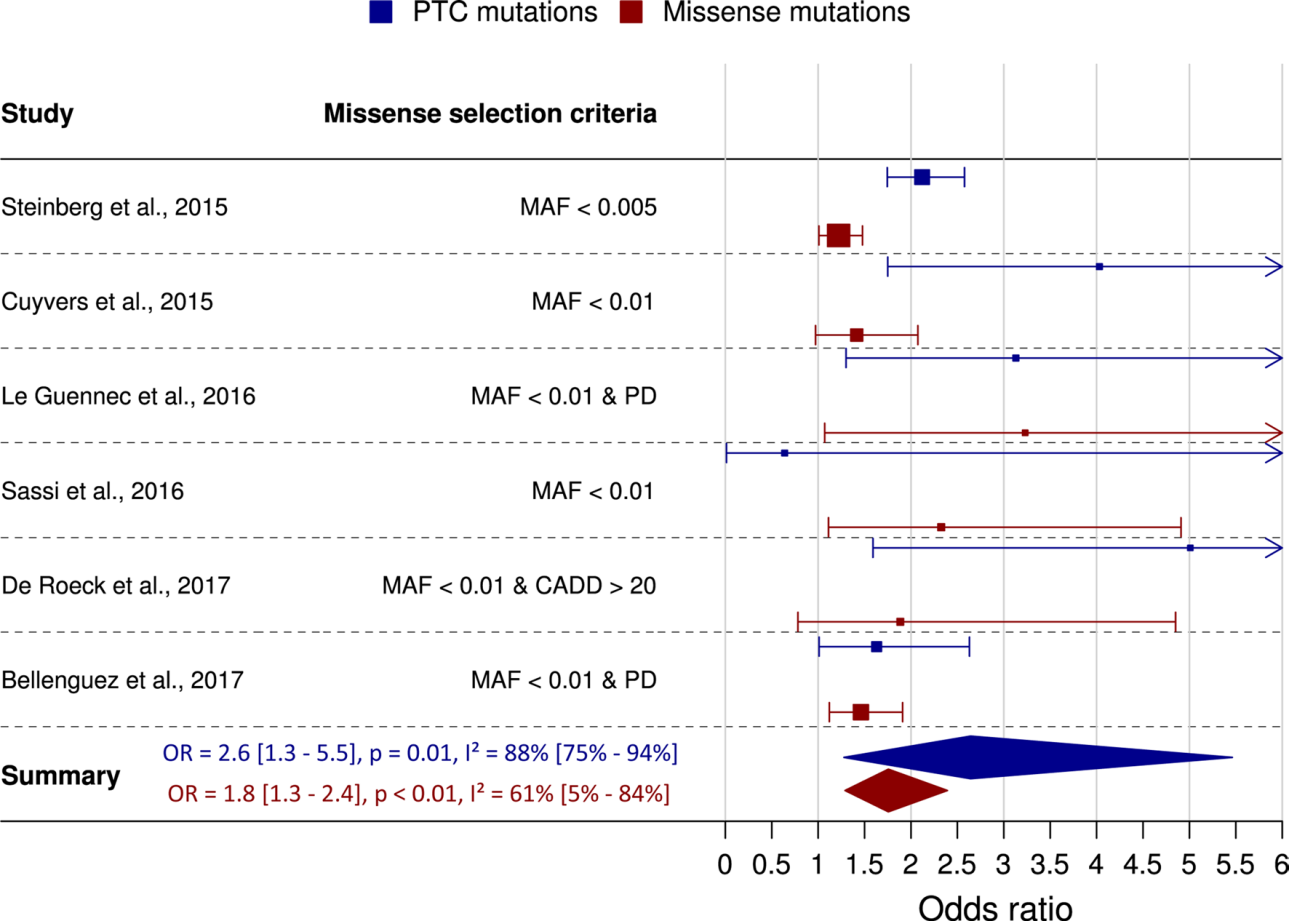
Meta-analysis of risk effects for *ABCA7* PTC (blue) and rare missense (red) variants from Caucasian AD case–control studies. Included studies had to meet the following criteria: more than 900 included individuals, NGS analysis of the complete *ABCA7* coding sequence, and odds ratios had to be inferable from the report. Of note, one African American study met these criteria, but was not included in this meta-analysis due to strong differences in PTC frequencies between Caucasian and African American cohorts [76]. Study-specific odds ratios (indicated with solid boxes, size of the box reflects the size of the cohort) are presented in a forest plot with 95% confidence intervals (CI; lines), as derived directly from the literature, or if not reported, inferred from published variant count data. The pooled effect estimate and 95%CI (indicated with diamonds) were generated using a random effects model incorporated in R-package meta [87]. Visualization was performed with R-package forestplot [88]. Studies used different criteria for reporting of missense variants: *MAF* minor allele frequency, *PD* predicted damaging by three in silico prediction programs (MutationTaster, SIFT, and Polyphen2), *CADD* phred-scaled combined annotation dependent depletion score, *OR* odds ratio with 95% CI, *p* random effects *p* value, *I*^2^ = *I*^2^ heterogeneity statistic with CI

Thirteen published studies have examined the associations of *ABCA7* PTC variants in AD–control cohorts [80, 81, 75, 71, 82, 83, 72, 76, 84–86, 77, 79]. All, but one, observed a higher frequency of heterozygous PTC variants in patients than controls; yet a large range of ORs is reported varying from approximately 1.4 [76] to 5 [85] in studies reporting an enrichment in patients. This relatively low 1.4 OR was reported in a study conducted on an African American cohort [76], and was mostly driven by a substantially weaker risk effect for common African American PTC p.Arg578 fs than its originally reported effect size (Table 1). Meta-analysis of six Caucasian studies that conducted a comprehensive NGS-based analysis of the entire *ABCA7* coding sequence on cohorts including > 900 individuals indicates a 2.6-fold PTC enrichment in AD patients compared to controls (Fig. 2, Table 1).

Cautious interpretation of these Caucasian risk estimates is warranted though since c.5570 + 5G > C (rs200538373), the most frequent Caucasian PTC variant, was not always included in calculations of study-specific effect estimates. This variant does not directly perturb the canonical sequence of a splice site, but causes the preferential usage of a cryptic splice donor site with a frameshift as a consequence [85, 77]. It is unclear whether this alternative splicing always occurs on the mutated allele, or if splicing is only partially affected. An in vitro assessment of c.5570 + 5G > C supports a consistent splicing effect of c.5570 + 5G > C [89]; whether this extrapolates to different human cell types needs to be verified.

According to the Genome Aggregation Database (gnomAD, accessed January 2019) [90], the carrier frequency of *ABCA7* PTC variants (including c.5570 + 5G > C) in the general European population is 2.1%, while most hypothesis-free studies report between 3.0 and 4.4% of AD patients carrying a PTC variant, with differences most likely driven by founder effects [81, 71, 83, 85, 77]. In African American individuals, a staggering 14.5% carries a PTC variant. This cumulative frequency further increases to 21.7% in AD patients [76]. This high PTC mutational frequency is primarily due to the common PTC variant p.Arg578 fs which is proposed to explain the African American GWAS signal at *ABCA7* (Table 1). With the exception of variants in canonical splice sites, c.5570 + 5G > C, and the *ABCA7* VNTR, no other *ABCA7* variants have been reported to affect splicing, but we note a lack of comprehensive studies, suggesting these carrier frequencies may be underestimated. Importantly, the observation of *ABCA7* PTC variants in control individuals and the high frequency of certain variants among African Americans indicates that *ABCA7* PTC variants per se are not sufficient to cause disease, but rather risk variants of intermediate to high penetrance. Identification of factors that modify the penetrance of these variants will be pivotal in understanding how *ABCA7* PTC variants affect AD risk.

### ABCA7 haploinsufficiency is the most plausible downstream mechanism for PTC variants

PTC variants can lead to the formation of truncated ABCA7 proteins, which, in theory, can underlie the AD risk-increasing effect by several mechanisms. First of all, truncated proteins may cause toxicity in the cell through a gain of function (GOF) effect. In this case, pathogenic mutations are expected to cluster in parts of the gene, affecting particular protein domains. Second, truncated proteins may present a dominant negative effect: an abolition of the function of proteins binding to the mutated protein; often observed in multimeric protein complexes. Third, the truncated proteins may no longer exert an effect, or can be subject to quick degradation, which leads to a loss-of-function (LOF). To avoid the first two deleterious scenarios, cells have a mechanism termed nonsense-mediated mRNA decay (NMD) to track down transcripts containing a PTC which are subsequently degraded prior to the formation of truncated proteins. Intervention through NMD results in LOF. In general, loss of one gene copy is tolerated; however, for some dosage-sensitive genes, losing half of the expression may cause disease, which is termed haploinsufficiency [91]. In the case of *ABCA7* in AD, heterozygous PTC variants are observed across the entire transcript without an apparent pathological enrichment to certain protein domains (Fig. 1), making a GOF effect to be an unlikely mechanism for all PTC variants. Furthermore, the ABCA7 protein channel is formed as a monomer [11], reducing the likelihood of a dominant negative effect. As such, *ABCA7* haploinsufficiency is the most plausible mechanism leading to increased AD risk. This mechanism would be in line with the previously discussed in vitro and in vivo evidence, and the effect of common *ABCA7* variants (in particular the *ABCA7* VNTR). Further research into this hypothesis, by studying *ABCA7* expression, however, has been limited and has proven to be difficult. Currently, we do not know which isoforms of the ABCA7 protein form a functional entity, nor do we know what the physiological levels of functional *ABCA7* are in different cell types and/or tissues. Large variance is observed in brain *ABCA7* mRNA and protein expression, both between PTC variant carriers and non-carriers, and within the group of PTC variant carriers [80, 71]. Further allele-specific analyses of these transcripts reveal that incomplete NMD (or NMD escape) of *ABCA7* transcripts harboring a PTC is common for most PTC variants and the abundance of these PTC transcripts can vary from 5% (strong mRNA degradation through NMD) to approximately 50% (almost no NMD degradation) [80, 75, 85]. Interestingly, long-read phasing of *ABCA7* cDNA revealed that for several PTC variants, the induced PTC could be removed from the transcript by alternative splicing (i.e. restoration of the reading frame by usage of a cryptic splice site, or removal of a nonsense variant through in-frame exon skipping). These “PTC rescue mechanisms” have the potential to increase the abundance of functional ABCA7 protein [85]. Varying NMD efficiency and PTC rescue splicing, however, explains only a fraction of *ABCA7* expression differences, and the effect on penetrance and clinical presentation remains to be explored. Further research into different *ABCA7* isoforms, the upstream factors regulating *ABCA7* expression, and correction for brain tissue composition (i.e. correction for the influx and activation of microglia) is necessary to understand when *ABCA7* haploinsufficiency kicks in and how this increases the risk for AD.

### The role of ABCA7 PTC variants on clinical phenotypes

**Fig. 3:**
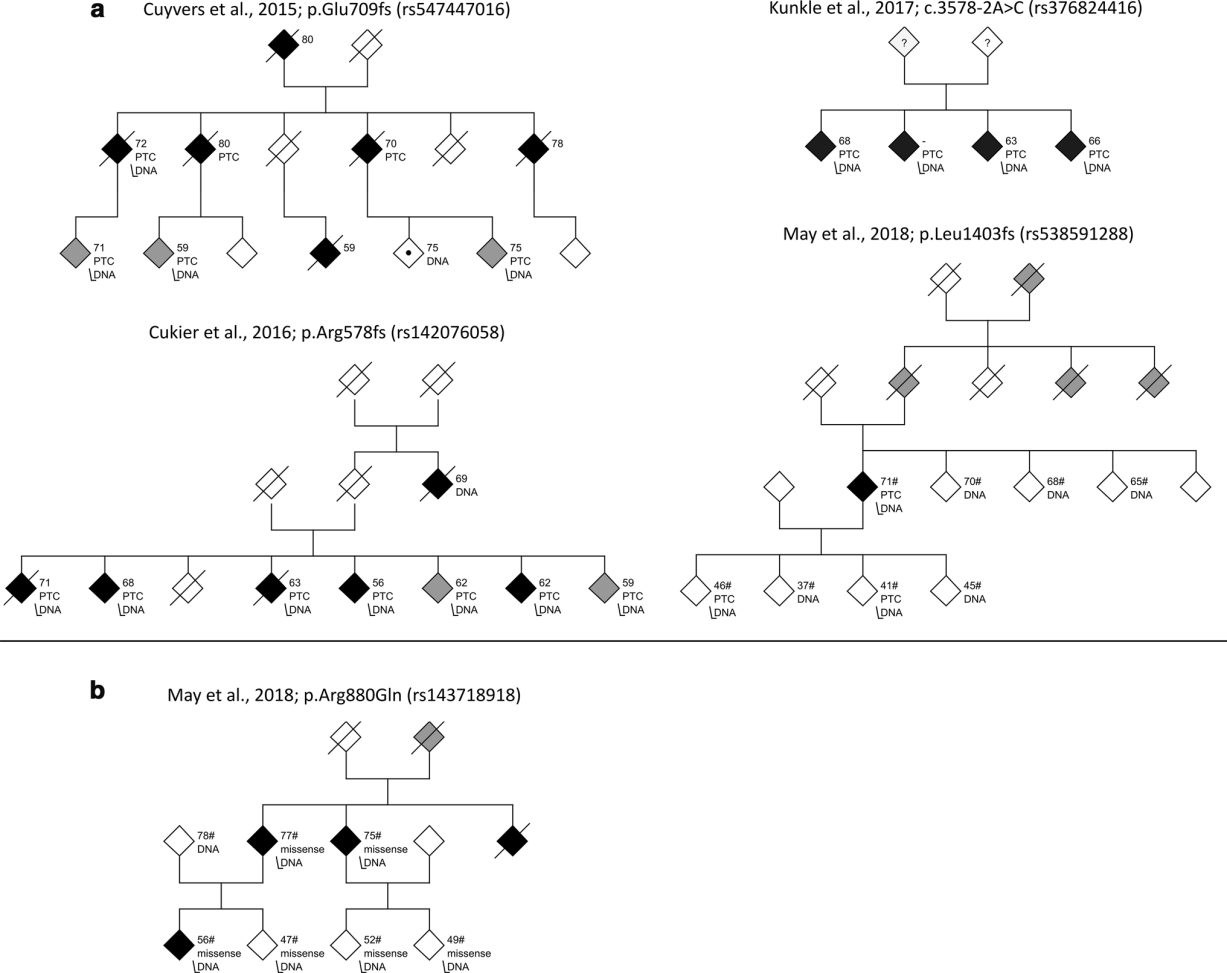
AD pedigrees reported to demonstrate potential segregation of *ABCA7* PTC (**a**) or missense (**b**) variants. Individuals are represented by diamonds, annotated according to their disease status: AD (black), MCI or suggestive cognitive decline (gray), Parkinson disease (diamond with dot), unaffected at the time of examination (white), and unknown (question mark). Striked through diamonds correspond to deceased individuals, numbers depict the age at onset, or age at examination (marked with #). PTC = this individual carries the PTC variant. Missense = this individual carries the missense variant. DNA = this individual had DNA available and was screened for carriership of the variant

Carrying an *ABCA7* PTC variant, in general, more than doubles the risk for developing AD, with local variation due to founder effects. Two reports show an increased proportion of positive familial history for AD patients with an *ABCA7* PTC compared to the overall patient cohort from which they originated [92, 85]. While familial clustering is in line with an increased penetrance of these variants, observation of segregation patterns mimicking autosomal dominant inheritance is exception rather than rule. Only four pedigrees have been reported so far; moreover, co-segregation in these pedigrees was neither significant nor clear for all carriers (Fig. 3) [75, 71, 72, 93].

Two studies examined whether *ABCA7* PTC variants could also affect the risk of other neurodegenerative conditions by comparing healthy controls to, respectively, 840 Parkinson disease (PD) patients [94] and a cohort of 381 brain autopsied non-AD dementia patients (enriched for vascular dementia, frontotemporal dementia, dementia with Lewy bodies, and progressive supranuclear palsy) [80]. A strong enrichment (OR = 4.9) of *ABCA7* PTC variants was observed in PD, albeit not reaching significance (*p* = 0.07) [94]. For the non-AD dementia cohort, a significant enrichment was observed [OR = 3.1 (1.2–7.7)] and this effect size was similar when compared to the enrichment of AD patients and controls [80]. Hence, these reports suggest that *ABCA7* PTC variants may play a general role in neurodegeneration, but more research with meticulously diagnosed patient cohorts is necessary.

Since AD GWAS were mainly based on LOAD patients, *ABCA7* was originally considered a LOAD risk gene. Studies investigating *ABCA7* PTC variants on the other hand have included a substantial number of EOAD patients and found an enrichment of PTC variants in these individuals as well [81, 92, 75, 83, 85]. While Bellenguez et al. observed slightly increased enrichment of *ABCA7* PTC variants in EOAD, compared to LOAD [81], other studies did not observe an effect of *ABCA7* PTC variants on onset age [75, 71]. Overall, *ABCA7* PTC variant carriers have a very wide onset age range, from 46 [83] to 90 years old [71], even within carriers of the same variant. While an onset age of 46 is very young for AD, onset ages for carriers of pathogenic mutations in causal genes, such as *PSEN1*, are generally lower [85]. The wide variation in onset ages is to be expected for (rare) risk variants.

Only few studies so far have investigated clinical characteristics of *ABCA7* PTC variant carriers other than onset age. In a series of 22 AD patients carrying an *ABCA7* PTC variant, all presented with a classical AD phenotype, with progressive memory impairment [92]. Neuropathological assessment of autopsied brains of PTC variant carriers was in accordance with typical AD [92, 85]. Additional research into the downstream phenotypic effects of PTC variants has been scarce. Only one study assessed a possible relation with amyloid deposition: Martiskainen et al. examined the effect of the c.5570 + 5G > C (rs200538373) risk allele on Aβ_40_ and Aβ_42_ levels in blood plasma of healthy individuals, but no associations were observed [95]. Nevertheless, the correlation between plasma amyloid levels and AD has been contradictory, and in general, only marginal differences are observed between patients and controls [96]. Further investigation of relevant biomarkers is, therefore, necessary.

### Enrichment of missense variants in Alzheimer’s disease

Analogous to PTC variants in *ABCA7*, other variant categories most likely influence the risk to develop AD. In particular, missense variants have the potential to destroy important protein domains, or they can interfere with protein stability and degradation; both of which can lead to a LOF effect. One study reported the co-segregation of missense variant p.Arg880Gln (rs143718918) with autosomal dominant AD (Fig. 3) [93]. Based on in silico prediction, this variant has a high chance to disrupt *ABCA7* function. With a Phred-scaled CADD score of 28.6, p.Arg880Gln is considered to be among the 0.14% most deleterious variants of the genome [41]. Further analysis of *ABCA7* missense variants has been limited, and no meta-analysis exists to date. Nevertheless, several NGS studies have reported the observed frequencies of missense variants in AD patients and controls [81, 71, 83, 85, 86, 77], albeit using different criteria to determine which variants are to be reported, potentially biasing downstream analyses. While all studies focus on rare variants, some studies only report variants with in silico-predicted deleterious effects on the *ABCA7* protein (Fig. 2). Meta-analysis of all published reports on missense variants in *ABCA7* suggests this class of variants also confers a 1.8-fold enrichment in AD patients (Table 1); however, the validity of these effect sizes has to be confirmed with additional research. On the one hand, they could have been artificially augmented due to different missense selection criteria across the studies. On the other hand, however, we may be underestimating the effect of missense variants on AD, since the direction of effect for all missense variants under study is considered to be equal. In reality, it is most likely that while some missense variants may be deleterious, others will have no effect, or may even protect from disease. Rare variant association testing can take these different effect directions into account (e.g. Sequence Kernel Association Test (SKAT) [97]), though they should be conducted on variants which are not selected based on predicted pathogenicity, and the underlying variants need to be reported with carrier frequencies for interpretation. Better understanding of the function and structure of ABCA7, as well as experimental assays to evaluate the effect of variants, are needed to enable classification of missense variants. A bidirectional effect is supported by common *ABCA7* missense variants: two studies observed significant protective effects for p.Gly215Ser (rs72973581, Table 1) [85, 86]. This residue is situated in the extracellular domain of *ABCA7*. Interestingly, the minor and protective serine amino acid is evolutionary conserved in several mammals, as well as the homologous human ABCA1 protein, which favors a functional effect of this missense variant.

### Methylation in the *ABCA7* locus affects Alzheimer’s disease

In addition to genetic variations, epigenetic modifications of DNA and DNA-bound histones can alter gene expression. One of the best-known and most studied modifications is methylation of cytosine nucleotides at position 5 of the pyrimidine ring. This 5-methylcytosine occurs most prominently in a cytosine:guanine (CpG) sequence. These motifs often cluster together in so-called CpG islands, and regions flanking these islands are termed CpG island shores. Historically, DNA methylation was mostly associated with gene expression silencing, yet increasing research shows that, depending on the genomic context, the effects of methylation are highly variable [98].

The role of methylation in AD was assessed at genome-wide scale within the frame of the Religious Order Study (ROS) and the Memory and Aging Project (MAP). DNA CpG methylation was examined in more than 700 gray-matter brain tissues from the dorsolateral prefrontal cortex. Of the 415,848 examined CpG dinucleotides, 71 reached genome-wide significance; two of which corresponded to AD GWAS loci: *BIN1* and *ABCA7* [99]. In-depth analysis of the *ABCA7* locus showed that three CpG sites (cg02308560, cg24402332, and cg04587220) associated with AD after correction for multiple testing (Fig. 1, Table 1). Methylation of each of these CpG dinucleotides correlated with a higher risk of developing AD [99, 100]. Additionally, 12 CpG sites in *ABCA7* correlated with Aβ load and 18 CpG sites correlated with tau tangle density [100]. Across all of these endophenotypes, cg02308560 and cg04587220 generally had the strongest associations, which were independent from *ABCA7* AD GWAS sentinel SNP genotypes [101, 99]. They are only 30 base pairs apart from each other and located approximately 5.6 kb downstream of the *ABCA7* gene, embedded in a CpG island which is part of a Polycomb repressed region situated in an intron of the *HMHA1* gene [100]. DNA methylation is an important regulator of Polycomb-mediated repression, which can further regulate the expression of nearby genes, such as *ABCA7* [102]. Overall, CpG methylation in *ABCA7*, however, did not seem to have strong effects on brain *ABCA7* mRNA expression [100].

In addition, Humphries et al. examined methylation in a smaller series of brain tissue samples from the temporal pole. For all 20 AD GWAS loci under study, the strongest effects were observed in the *ABCA7* locus. More specifically, a CpG island shore, located in exon 12 and intron 12 of *ABCA7* (chr19:1045074–1045679, Fig. 1), was hypomethylated in LOAD when compared to healthy control brains, and brains of patients with dementia with Lewy bodies (Table 1) [103]. Due to usage of different methylation detection techniques, this CpG island shore was not assessed in the previously discussed methylation studies.

Not all AD-related methylation studies, however, identified significant changes in the *ABCA7* locus. Lunnon et al. performed a (smaller sample size) genome-wide methylation assessment using the same methylation array as described above, but in different brain regions (entorhinal cortex, cerebellum, superior temporal gyrus, and prefrontal cortex) and blood. None of the top 100 associated methylation markers across these tissues was positioned in the *ABCA7* locus [104]. Additionally, Yamazaki et al. analyzed methylation of five CpG sites in the promotor region of *ABCA7* in peripheral blood samples, but found no differential methylation between patients and controls [105]. Hence, while several independent studies have reported a role for CpG methylation of *ABCA7* in AD risk, the mode of action remains to be elucidated. More research, accounting for factors such as brain regions and tissue composition, could provide more clarity into the downstream effects of *ABCA7* methylation.

### Differences in *ABCA7* splicing between patients and controls

In addition to previously discussed DNA-based findings, alterations on the *ABCA7* mRNA level have been linked to AD as well. Based on cDNA cloning and northern blotting, early literature on *ABCA7* described two isoforms of *ABCA7*: full-length *ABCA7* (“type I”, RefSeq ID: NM_019112, GENCODE ID: ENST00000263094.10_1) and a splice variant (“type II”). The type II isoform originates from alternative splicing of the *ABCA7* pre-mRNA which does not remove intron 6 from the coding sequence. This introduces a PTC, which is then followed by a novel in-frame start codon. The coding sequence initiating at this novel start codon and containing the 41 downstream exons corresponds to type II *ABCA7* (resembles GENCODE ID ENST00000435683.6_1) [106]. The abundance of both isoforms was highly variable between human tissues. In vitro experiments further showed that both isoforms translate into proteins with different cellular localization. Type I resided both on the plasma membrane and in intracellular compartments. Type II on the other hand was predominantly localized in the endoplasmic reticulum. Finally, in contrast to type I, type II did not show efficient apoA-I-mediated lipid release [106].

As a consequence of large-scale RNA-sequencing studies and public transcript repositories such as GENCODE [107], we now know that the transcriptional landscape of *ABCA7* is far more complex. According to GENCODE version 28 [Accessed January 2019], 19 different transcripts are allocated to the *ABCA7* gene. Nevertheless, it is unknown which of these are translated into proteins and what their functional relevance is. These known transcripts, however, are only the tip of the iceberg. Two reports analyzed splicing patterns of a dozen of *ABCA7* exons in depth and identified alternative splicing correlated with *ABCA7* VNTR lengths, and found splicing events which could potentially rescue a deleterious PTC variant. Incidentally, naturally occurring alternative splicing was observed in virtually all exons [85, 73], which gives rise to a myriad of potential transcripts. Large deviations from the full-length *ABCA7* transcript are presumably detrimental to its functions, since the bulk of the *ABCA7* transcript is essential to form an ABCA7 protein with a transmembrane pore and ATP-hydrolyzing NBD domains (Fig. 1). Yet, more research is needed to support this hypothesis and to understand the consequences of smaller transcript alterations.

Due to the relatively low expression of *ABCA7*, and consequently low sequencing read counts in RNA-sequencing experiments, it is not trivial to study alternative splicing in *ABCA7*. Nevertheless, in the largest AD meta-analyzed transcriptome-wide association study (TWAS) to date, significant association was found between *ABCA7* splicing—measured in the dorsolateral prefrontal cortex—and AD [108]. These findings were further corroborated by another study analyzing RNA from the temporal lobe brain region, in which differences in *ABCA7* splicing between LOAD and controls were observed [103]. The strongest TWAS alternative splicing signal mapped to the 3′ end of the *ABCA7* transcript, which is most likely the result of technical limitations. The algorithms used for characterization of alternative splicing require a minimum of supporting sequencing reads, and RNA-sequencing coverage is often enriched in the 3′ transcript regions. Hence, due to the low expression of *ABCA7*, this alternative splicing analysis was probably most powerful in the 3′ end [109]. Further research with higher sequencing depth, or enrichment of *ABCA7* transcripts could provide more clarity.

### Potential inverse *ABCA7* correlation with cancer

Interestingly, while evidence is limited compared to neurodegenerative diseases, *ABCA7* has also been linked to cancer. *ABCA7* expression is increased in ovarian cancer tissues [110, 111], and this upregulation has been shown to accelerate the epithelial-to-mesenchymal transition, a process involved in the initiation of metastasis. In vitro knockdown of *ABCA7* in ovarian cancer cells reversed markers of epithelial-to-mesenchymal transition, suggesting that *ABCA7* is actively involved in this process. Furthermore, ovarian cancer patients with high *ABCA7* mRNA expression levels, have a poorer survival rate than patients with low *ABCA7* expression [111]. In addition to ovarian cancer, increased expression of *ABCA7* was observed in pancreatic ductal adenocarcinoma tumors [112]. Melanoma cell lines on the other hand showed lower expression levels in comparison with melanocytes [113].

Hence, while more research is needed, it seems that an increase of *ABCA7* expression may lead to a higher risk of specific cancer-type development, which is opposite to *ABCA7* loss-of-function as a pathomechanism of AD. This observation is in line with several epidemiological reports observing an inverse comorbidity between central nervous system (CNS) disorders and cancer [114]. Biological evidence further supports this finding; hence, this is most likely a true association rather than an indirect effect caused by a survival bias. Notably, inverse up- and downregulation of genes in CNS disorders and cancer have been observed [115], and several genetic loci have shown opposite effects in AD and cancer GWAS studies [116]. Further research into *ABCA7* inverse comorbidities is warranted to understand the implications of *ABCA7* expression as a possible therapeutic target for dementia.

### Summary and future prospects

**Fig. 4:**
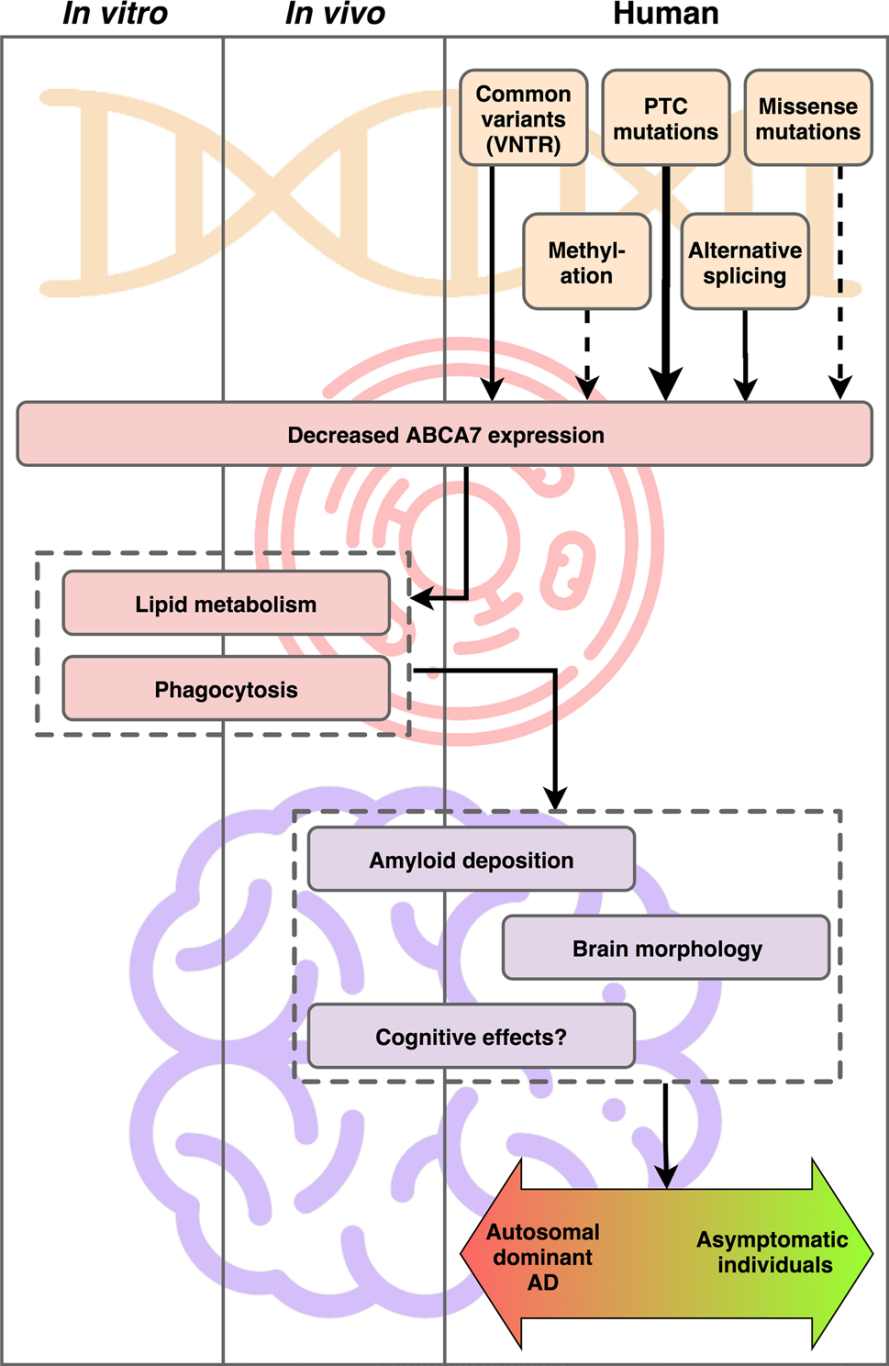
Overview of current *ABCA7* AD-related evidence from in vitro model systems, in vivo model systems, and human-based studies. A possible pathogenic pathway is shown: alterations on the *ABCA7* DNA (transcription) level (orange) can affect cellular homeostasis and functions (red), leading to changes in the brain and potential neurodegenerative disease (purple). The arrow style (ranging from dashed lines to thick full lines) corresponds to the strength of current evidence supporting that connection

In the past 8 years, human-based studies have demonstrated numerous *ABCA7*-related associations with AD; including four GWAS SNPs, 52 PTC variants, rare missense variants, a protective common missense variant, a tandem repeat, CpG methylation, and alternative splicing. As such, compelling evidence supports the involvement of *ABCA7* in AD pathophysiology (Fig. 4). Combined, these *ABCA7* alterations contribute substantially to the occurrence of both EOAD and LOAD patients (e.g. the heritability of liability attributable to expanded *ABCA7* VNTRs and PTC variants in the Caucasian cohort is approximately 4.5% [117]) Furthermore, in specific cases, PTC variants have been observed to achieve high disease penetrance, leading to inheritance patterns resembling autosomal dominancy. Nevertheless, cautious interpretation is needed regarding the effects of *ABCA7* alterations on the phenotype. Overall, variable disease penetrance and onset ages are observed, and it is currently not known whether *ABCA7* variants are AD specific or if they contribute to other dementia phenotypes as well. Hence, a better understanding of *ABCA7* modifiers is warranted. An important avenue of investigation in this regard, and potentially the biggest caveat in *ABCA7* research to date, is the expression of (different isoforms of) *ABCA7*. AD-associated markers in *ABCA7*, as well as in vitro and in vivo experiments, point towards a decrease of *ABCA7* as the main pathological mechanism. As a consequence, varying *ABCA7* expression levels could modify the phenotype. Moreover, targeting *ABCA7* transcription or splicing to increase functional ABCA7 expression levels could be a valuable therapeutic approach. However, based on the Accelerating Medicines Partnership—Alzheimer’s Disease (AMP-AD) consortium which includes the currently largest collaborative post-mortem brain RNA-sequencing project, no significant differential expression of *ABCA7* is observed between patients and controls [https://agora.ampadportal.org; accessed January 2019]. This is further corroborated by a recent meta-analysis of AD brain transcriptomics data [118].

Several hypotheses could underlie this situation. First, perturbation of *ABCA7* expression is potentially not a canonical pathological pathway of AD, and therefore, not observed in a (underpowered) heterogeneous population of AD patients. In this scenario, *ABCA7* haploinsufficiency could give rise to a molecular subtype of AD, with potentially different clinical and therapeutic characteristics. Hence, patient stratification based on *ABCA7* variant status could be advantageous, both to detect decreased *ABCA7* expression and for downstream investigations. Second, *ABCA7* downregulation due to deleterious missense variants (e.g. by causing misfolding and/or increased protein degradation) are not observed on a transcript level. *ABCA7* mRNA downregulation is also partially masked for PTC variants because of NMD escape and PTC rescue splicing. Additionally, differences in splicing (e.g. as observed with different *ABCA7* VNTR lengths) are generally not detected in conventional *ABCA7* quantification. Third, decreased *ABCA7* may contribute to AD pathology in the early stages of disease; however, it is possible that at later stages (which are measured in post-mortem brain), *ABCA7* is upregulated as a compensatory mechanism in AD brains; e.g. to increase phagocytic clearance of amyloid plaques. Last, *ABCA7* expression is likely to differ between cell (sub-) types and cellular states (e.g. ramified versus activated microglia), and this cell type composition in turn varies between patient and control brains which biases overall expression analyses. Additionally, it is not completely understood in which cell type(s) and brain regions *ABCA7* exerts its (AD-related) functions.

Hence, to provide an answer to these hypotheses, there is a need for: (1) a better understanding of the function of ABCA7 and in which cell types, brain regions, and disease stages it plays a role; (2) functional studies and assays which can differentiate deleterious variants and splicing events from functional *ABCA7* forms; (3) more sensitive assays for both the mRNA and protein level which are capable of detecting and quantifying different isoforms of the lowly expressed *ABCA7* gene. Recent developments in the sequencing field can provide solutions. On the one hand, single-cell sequencing can give novel insights into the cell- and tissue-specificity of *ABCA7*. While not always trivial for (post-mortem) brain tissue, adaptations to this technique such as single nuclei sequencing [119] and spatial transcriptomics [120] can provide an alternative. On the other hand, long-read sequencing techniques can sequence entire mRNA molecules from start to end and, therefore, provide a better understanding and quantification of all possible *ABCA7* transcripts [121, 122]. For both single-cell and long-read sequencing, high sequencing depth is necessary to detect the relatively lowly expressed *ABCA7* transcripts, or specific capture of *ABCA7* mRNA prior to sequencing needs to occur. Other developments which may benefit our understanding of *ABCA7* include better in vitro models based on induced pluripotent stem cells (iPSCs), which can be differentiated into neuronal and glial cell types of interest [123]. These models can be extended to three-dimensional organoids to encompass the interplay between different brain cells [124]. Furthermore, improvements in the field of cryogenic electron microscopy have recently allowed to determine the protein structure of ABCA1 [125]. Hence, a similar approach should be feasible for ABCA7, which would enable improved in silico predictions of *ABCA7* functions and effects of variants and splicing.

Currently, to determine methylation, or genotype sentinel SNPs, rare variants, and tandem repeats, different technologies are used (e.g. bisulfite sequencing or methylation arrays, SNP arrays, NGS and Southern blotting). Consequently, these markers are often studied independently, leading to a missed understanding of their combined effects on AD. One way to resolve this issue would be the usage of long-read sequencing, which enables simultaneous detection of SNPs, structural variants such as tandem repeats, and DNA methylation. Recently, we showed that robust whole-genome long-read sequencing is possible on PromethION (Oxford Nanopore Technologies), and accurate detection of *ABCA7* VNTR alleles, including expansions, was achieved [74]. With continuous improvements to single-nucleotide accuracy, these whole-genome long-read sequencing techniques will hopefully soon enable simultaneous detection of all DNA-related markers in *ABCA7* and other AD loci, with clinical-grade quality [126]. In addition, this would enable discovery of other AD-associated structural variants and epigenetic markers, and would facilitate easier transition from research to *ABCA7* genetic screening in the clinic.

## References

[1] Winblad B, Amouyel P, Andrieu S, Ballard C, Brayne C, Brodaty H (2016). Defeating Alzheimer’s disease and other dementias: a priority for European science and society. Lancet Neurol.

[2] Jack CR, Petersen RC, Xu YC, O’Brien PC, Smith GE, Ivnik RJ (1999). Prediction of AD with MRI-based hippocampal volume in mild cognitive impairment. Neurology.

[3] Cacace R, Sleegers K, Van Broeckhoven C (2016). Molecular genetics of early-onset Alzheimer disease revisited. Alzheimer’s Dement.

[4] Cuyvers E, Sleegers K (2016). Genetic variations underlying Alzheimer’s disease: evidence from genome-wide association studies and beyond. Lancet Neurol.

[5] Abe-Dohmae S, Ueda K, Yokoyama S (2006). ABCA7, a molecule with unknown function. FEBS Lett.

[6] Aikawa T, Holm ML, Kanekiyo T (2018). ABCA7 and pathogenic pathways of Alzheimer’s disease. Brain Sci.

[7] Li H, Karl T, Garner B (2015). Understanding the function of ABCA7 in Alzheimer’s disease. Biochem Soc Trans.

[8] Tanaka N, Abe-Dohmae S, Iwamoto N, Yokoyama S (2011). Roles of ATP-binding cassette transporter A7 in cholesterol homeostasis and host defense system. J Atheroscler Thromb.

[9] Zhao Q-F, Wan Y, Wang H-F, Sun F-R, Hao X-K, Tan M-S (2016). ABCA7 genotypes confer Alzheimer’s disease risk by modulating amyloid-β pathology. J Alzheimers Dis.

[10] LaFerla FM, Green KN (2012). Animal models of Alzheimer disease. Cold Spring Harb Perspect Med.

[11] Kaminski WE, Orsó E, Diederich W, Klucken J, Drobnik W, Schmitz G (2000). Identification of a novel human sterol-sensitive ATP-binding cassette transporter (ABCA7). Biochem Biophys Res Commun.

[12] Dean M, Annilo T (2005). Evolution of the Atp-binding cassette (Abc) transporter superfamily in vertebrates. Ann Rev Genom Hum Genet.

[13] Berman HM, Westbrook J, Feng Z, Gilliland G, Bhat TN, Weissig H (2000). The protein data bank. Nucleic Acids Res.

[14] Kim WS, Fitzgerald ML, Kang K, Okuhira K, Bell SA, Manning JJ (2005). Abca7 null mice retain normal macrophage phosphatidylcholine and cholesterol efflux activity despite alterations in adipose mass and serum cholesterol levels. J Biol Chem.

[15] Kim WS, Guillemin GJ, Glaros EN, Lim CK, Garner B (2006). Quantitation of ATP-binding cassette subfamily—a transporter gene expression in primary human brain cells. NeuroReport.

[16] Zhang Y, Chen K, Sloan SA, Bennett ML, Scholze AR, O’Keeffe S (2014). An RNA-sequencing transcriptome and splicing database of glia, neurons, and vascular cells of the cerebral cortex. J Neurosci.

[17] Zhang Y, Sloan SA, Clarke LE, Caneda C, Plaza CA, Blumenthal PD (2016). Purification and characterization of progenitor and mature human astrocytes reveals transcriptional and functional differences with mouse. Neuron.

[18] Krzywinski M, Schein J, Birol I, Connors J, Gascoyne R, Horsman D (2009). Circos: an information aesthetic for comparative genomics. Genome Res.

[19] Abe-Dohmae S, Ikeda Y, Matsuo M, Hayashi M, Okuhira K, Ueda K (2004). Human ABCA7 supports apolipoprotein-mediated release of cellular cholesterol and phospholipid to generate high density lipoprotein. J Biol Chem.

[20] Hayashi M, Abe-Dohmae S, Okazaki M, Ueda K, Yokoyama S (2005). Heterogeneity of high density lipoprotein generated by ABCA1 and ABCA7. J Lipid Res.

[21] Linsel-Nitschke P, Jehle AW, Shan J, Cao G, Bacic D, Lan D (2005). Potential role of ABCA7 in cellular lipid efflux to apoA-I. J Lipid Res.

[22] Wang N, Lan D, Gerbod-Giannone M, Linsel-Nitschke P, Jehle AW, Chen W (2003). ATP-binding cassette transporter A7 (ABCA7) binds apolipoprotein A-I and mediates cellular phospholipid but not cholesterol efflux. J Biol Chem.

[23] Iwamoto N, Abe-Dohmae S, Sato R, Yokoyama S (2006). ABCA7 expression is regulated by cellular cholesterol through the SREBP2 pathway and associated with phagocytosis. J Lipid Res.

[24] Sakae N, Liu C-C, Shinohara M, Frisch-Daiello J, Ma L, Yamazaki Y (2016). ABCA7 deficiency accelerates amyloid-beta generation and Alzheimer’s neuronal pathology. J Neurosci.

[25] Nowyhed HN, Chandra S, Kiosses W, Marcovecchio P, Andary F, Zhao M (2017). ATP binding cassette transporter ABCA7 regulates NKT cell development and function by controlling CD1d expression and lipid raft content. Sci Rep.

[26] Wu YC, Horvitz HR (1998). The *C. elegans* cell corpse engulfment gene ced-7 encodes a protein similar to ABC transporters. Cell.

[27] Tanaka N, Abe-Dohmae S, Iwamoto N, Fitzgerald ML, Yokoyama S (2010). Helical apolipoproteins of high-density lipoprotein enhance phagocytosis by stabilizing ATP-binding cassette transporter A7. J Lipid Res.

[28] Fu Y, Hsiao J-HT, Paxinos G, Halliday GM, Kim WS (2016). ABCA7 mediates phagocytic clearance of amyloid-β in the brain. J Alzheimers Dis.

[29] Kim WS, Li H, Ruberu K, Chan S, Elliott DA, Low JK (2013). Deletion of Abca7 increases cerebral amyloid-β accumulation in the J20 mouse model of Alzheimer’s disease. J Neurosci.

[30] Kanekiyo T, Bu G (2014). The low-density lipoprotein receptor-related protein 1 and amyloid-β clearance in Alzheimer’s disease. Front Aging Neurosci.

[31] Lamartinière Y, Boucau M-C, Dehouck L, Krohn M, Pahnke J, Candela P (2018). ABCA7 downregulation modifies cellular cholesterol homeostasis and decreases amyloid-β peptide efflux in an in vitro model of the blood-brain barrier. J Alzheimer’s Dis.

[32] Satoh K, Abe-Dohmae S, Yokoyama S, George-Hyslop P, Fraser PE (2015). ATP-binding cassette transporter A7 (ABCA7) loss of function alters Alzheimer amyloid processing. J Biol Chem.

[33] Logge W, Cheng D, Chesworth R, Bhatia S, Garner B, Kim WS (2012). Role of Abca7 in mouse behaviours relevant to neurodegenerative diseases. PLoS One.

[34] Harold D, Abraham R, Hollingworth P, Sims R, Gerrish A, Hamshere ML (2009). Genome-wide association study identifies variants at CLU and PICALM associated with Alzheimer’s disease. Nat Genet.

[35] Hollingworth P, Harold D, Sims R, Gerrish A, Lambert J-C, Carrasquillo MM (2011). Common variants at ABCA7, MS4A6A/MS4A4E, EPHA1, CD33 and CD2AP are associated with Alzheimer’s disease. Nat Genet.

[36] Lambert J-C, Heath S, Even G, Campion D, Sleegers K, Hiltunen M (2009). Genome-wide association study identifies variants at CLU and CR1 associated with Alzheimer’s disease. Nat Genet.

[37] Lambert J-C, Ibrahim-Verbaas CA, Harold D, Naj AC, Sims R, Bellenguez C (2013). Meta-analysis of 74,046 individuals identifies 11 new susceptibility loci for Alzheimer’s disease. Nat Genet.

[38] Naj AC, Jun G, Beecham GW, Wang L, Vardarajan BN, Buros J (2011). Common variants at MS4A4/MS4A6E, CD2AP, CD33 and EPHA1 are associated with late-onset Alzheimer’s disease. Nat Genet.

[39] Reitz C, Wang L, Lin C, Larson EB, Graff-radford NR, Evans D (2013). Variants in the ATP-binding cassette transporter (ABCA7), apolipoprotein E and the risk of late-onset Alzheimer disease in African Americans. JAMA.

[40] Seshadri S, Fitzpatrick AL, Ikram MA, DeStefano AL, Gudnason V, Boada M (2010). Genome-wide analysis of genetic loci associated with Alzheimer disease. JAMA.

[41] Rentzsch P, Witten D, Cooper GM, Shendure J, Kircher M (2018). CADD: predicting the deleteriousness of variants throughout the human genome. Nucleic Acids Res.

[42] Machiela MJ, Chanock SJ (2015). LDlink: a web-based application for exploring population-specific haplotype structure and linking correlated alleles of possible functional variants. Bioinformatics.

[43] Almeida JFF, dos Santos LR, Trancozo M, de Paula F (2018). Updated meta-analysis of BIN1, CR1, MS4A6A, CLU, and ABCA7 variants in Alzheimer’s disease. J Mol Neurosci.

[44] Shulman JM, Chen K, Keenan BT, Chibnik LB, Fleisher A, Thiyyagura P (2013). Genetic susceptibility for Alzheimer disease neuritic plaque pathology. JAMA Neurol.

[45] Hughes TM, Lopez OL, Evans RW, Kamboh MI, Williamson JD, Klunk WE (2014). Markers of cholesterol transport are associated with amyloid deposition in the brain. Neurobiol Aging.

[46] Apostolova LG, Risacher SL, Duran T, Stage EC, Goukasian N, West JD (2018). Associations of the top 20 Alzheimer disease risk variants with brain amyloidosis. JAMA Neurol.

[47] Ma F, Zong Y, Wang H, Li J, Cao X, Tan L (2018). ABCA7 genotype altered Aβ levels in cerebrospinal fluid in Alzheimer’s disease without dementia. Ann Transl Med.

[48] Ramirez LM, Goukasian N, Porat S, Hwang KS, Eastman JA, Hurtz S (2016). Common variants in ABCA7 and MS4A6A are associated with cortical and hippocampal atrophy. Neurobiol Aging.

[49] Roshchupkin GV, Adams HH, van der Lee SJ, Vernooij MW, van Duijn CM, Uitterlinden AG (2016). Fine-mapping the effects of Alzheimer’s disease risk loci on brain morphology. Neurobiol Aging.

[50] Stage E, Duran T, Risacher SL, Goukasian N, Do TM, West JD (2016). The effect of the top 20 Alzheimer disease risk genes on gray-matter density and FDG PET brain metabolism. Alzheimer’s Dement Diagn, Assess Dis Monit.

[51] Sinha N, Reagh ZM, Tustison NJ, Berg CN, Shaw A, Myers CE (2018). ABCA7 risk variant in healthy older African Americans is associated with a functionally isolated entorhinal cortex mediating deficient generalization of prior discrimination training. Hippocampus.

[52] Wachinger C, Nho K, Saykin AJ, Reuter M, Rieckmann A (2018). A longitudinal imaging genetics study of neuroanatomical asymmetry in Alzheimer’s disease. Biol Psychiatry.

[53] Karch CM, Jeng AT, Nowotny P, Cady J, Cruchaga C, Goate AM (2012). Expression of novel Alzheimer’s disease risk genes in control and Alzheimer’s disease brains. PLoS One.

[54] Engelman CD, Koscik RL, Jonaitis EM, Okonkwo OC, Hermann BP, La Rue A (2013). Interaction between two cholesterol metabolism genes influences memory: findings from the Wisconsin registry for Alzheimer’s prevention. J Alzheimer’s Dis.

[55] Carrasquillo MM, Khan QUA, Murray ME, Krishnan S, Aakre J, Pankratz VS (2014). Late-onset Alzheimer disease genetic variants in posterior cortical atrophy and posterior AD. Neurology.

[56] Carrasquillo MM, Crook JE, Pedraza O, Thomas CS, Pankratz VS, Allen M (2015). Late-onset Alzheimer’s risk variants in memory decline, incident mild cognitive impairment, and Alzheimer’s disease. Neurobiol Aging.

[57] Nettiksimmons J, Tranah G, Evans DS, Yokoyama JS, Yaffe K (2016). Gene-based aggregate SNP associations between candidate AD genes and cognitive decline. Age (Omaha).

[58] Schott JM, Crutch SJ, Carrasquillo MM, Uphill J, Shakespeare TJ, Ryan NS (2016). Genetic risk factors for the posterior cortical atrophy variant of Alzheimer’s disease. Alzheimer’s Dement.

[59] Andrews SJ, Das D, Anstey KJ, Easteal S (2017). Late onset Alzheimer’s disease risk variants in cognitive decline: the PATH through life study. J Alzheimers Dis.

[60] Monsell SE, Mock C, Fardo DW, Bertelsen S, Cairns NJ, Roe CM (2017). Genetic comparison of symptomatic and asymptomatic persons with Alzheimer disease neuropathology. Alzheimer Dis Assoc Disord.

[61] Hohman TJ, Koran ME, Thornton-Wells T, Alzheimer’s Neuroimaging Initiative (2013). Epistatic genetic effects among Alzheimer’s candidate genes. PLoS One.

[62] Frisoni GB, Fox NC, Jack CR, Scheltens P, Thompson PM (2010). The clinical use of structural MRI in Alzheimer disease. Nat Rev Neurol.

[63] Vivot A, Glymour MM, Tzourio C, Amouyel P, Chêne G, Dufouil C (2015). Association of Alzheimer’s related genotypes with cognitive decline in multiple domains: results from the three-city Dijon study. Mol Psychiatry.

[64] Ober C, Vercelli D (2011). Gene-environment interactions in human disease: nuisance or opportunity?. Trends Genet.

[65] Crutch SJ, Lehmann M, Schott JM, Rabinovici GD, Rossor MN, Fox NC (2012). Posterior cortical atrophy. Lancet Neurol.

[66] Logue MW, Schu M, Vardarajan BN, Farrell J, Lunetta KL, Jun G (2014). Search for age-related macular degeneration risk variants in Alzheimer disease genes and pathways. Neurobiol Aging.

[67] Kjeldsen EW, Tybjærg-Hansen A, Nordestgaard BG, Frikke-Schmidt R (2018). ABCA7 and risk of dementia and vascular disease in the Danish population. Ann Clin Transl Neurol.

[68] Vasquez JB, Fardo DW, Estus S (2013). ABCA7 expression is associated with Alzheimer’s disease polymorphism and disease status. Neurosci Lett.

[69] Allen M, Zou F, Chai HS, Younkin CS, Crook J, Pankratz VS (2012). Novel late-onset Alzheimer disease loci variants associate with brain gene expression. Neurology.

[70] Bamji-Mirza M, Li Y, Najem D, Liu QY, Walker D, Lue L-F (2016). Genetic variations in ABCA7 can increase secreted levels of amyloid-β40 and amyloid-β42 peptides and ABCA7 transcription in cell culture models. J Alzheimer’s Dis.

[71] Cuyvers E, De Roeck A, Van den Bossche T, Van Cauwenberghe C, Bettens K, Vermeulen S (2015). Mutations in ABCA7 in a Belgian cohort of Alzheimer’s disease patients: a targeted resequencing study. Lancet Neurol.

[72] Kunkle BW, Carney RM, Kohli MA, Naj AC, Hamilton-Nelson KL, Whitehead PL (2017). Targeted sequencing of ABCA7 identifies splicing, stop-gain and intronic risk variants for Alzheimer disease. Neurosci Lett.

[73] De Roeck A, Duchateau L, Van Dongen J, Cacace R, Bjerke M, Van den Bossche T (2018). An intronic VNTR affects splicing of ABCA7 and increases risk of Alzheimer’s disease. Acta Neuropathol.

[74] De Roeck A, De Coster W, Bossaerts L, Cacace R, De Pooter T, Van Dongen J, et al. (2018) Accurate characterization of expanded tandem repeat length and sequence through whole genome long-read sequencing on PromethION. bioRxiv. 10.1101/439026 10.1186/s13059-019-1856-3 PMC6857246 31727106

[75] Cukier HN, Kunkle BW, Vardarajan BN, Rolati S, Hamilton-Nelson KL, Kohli MA (2016). ABCA7 frameshift deletion associated with Alzheimer disease in African Americans. Neurol Genet.

[76] Logue MW, Lancour D, Farrell J, Simkina I, Fallin MD, Lunetta KL (2018). Targeted sequencing of Alzheimer disease genes in African Americans implicates novel risk variants. Front Neurosci.

[77] Steinberg S, Stefansson H, Jonsson T, Johannsdottir H, Ingason A, Helgason H (2015). Loss-of-function variants in ABCA7 confer risk of Alzheimer’s disease. Nat Genet.

[78] Chen JA, Wang Q, Davis-Turak J, Li Y, Karydas AM, Hsu SC (2015). A multiancestral genome-wide exome array study of Alzheimer Disease, frontotemporal dementia, and progressive supranuclear palsy. JAMA Neurol.

[79] Vardarajan BN, Ghani M, Kahn A, Sheikh S, Sato C, Barral S (2015). Rare coding mutations identified by sequencing of Alzheimer disease genome-wide association studies loci. Ann Neurol.

[80] Allen M, Lincoln SJ, Corda M, Watzlawik JO, Carrasquillo MM, Reddy JS (2017). *ABCA7* loss-of-function variants, expression, and neurologic disease risk. Neurol Genet.

[81] Bellenguez C, Charbonnier C, Grenier-Boley B, Quenez O, Le Guennec K, Nicolas G (2017). Contribution to Alzheimer’s disease risk of rare variants in TREM2, SORL1, and ABCA7 in 1779 cases and 1273 controls. Neurobiol Aging.

[82] Del-Aguila JL, Fernández MV, Jimenez J, Black K, Ma S, Deming Y (2015). Role of ABCA7 loss-of-function variant in Alzheimer’s disease: a replication study in European-Americans. Alzheimers Res Ther.

[83] Le Guennec K, Nicolas G, Quenez O, Charbonnier C, Wallon D, Bellenguez C (2016). ABCA7 rare variants and Alzheimer disease risk. Neurology.

[84] Patel T, Brookes KJ, Turton J, Chaudhury S, Guetta-Baranes T, Guerreiro R (2018). Whole-exome sequencing of the BDR cohort: evidence to support the role of the PILRA gene in Alzheimer’s disease. Neuropathol Appl Neurobiol.

[85] De Roeck A, Van den Bossche T, van der Zee J, Verheijen J, De Coster W, Van Dongen J (2017). Deleterious ABCA7 mutations and transcript rescue mechanisms in early onset Alzheimer’s disease. Acta Neuropathol.

[86] Sassi C, Nalls MA, Ridge PG, Gibbs JR, Ding J, Lupton MK (2016). ABCA7 p. G215S as potential protective factor for Alzheimer’s disease. Neurobiol Aging.

[87] Schwarzer G (2007). meta: an R package for meta-analysis. R News.

[88] Gordon M, Lumley T (2017) forestplot: advanced forest plot using “grid” graphics. https://cran.r-project.org/package=forestplot

[89] Vasquez JB, Simpson JF, Harpole R, Estus S (2017). Alzheimer’s disease genetics and ABCA7 splicing. J Alzheimers Dis.

[90] Lek M, Karczewski KJ, Minikel EV, Samocha KE, Banks E, Fennell T (2016). Analysis of protein-coding genetic variation in 60,706 humans. Nature.

[91] Strachan Tom, Read Andrew P (2018). Human Molecular Genetics.

[92] Van den Bossche T, Sleegers K, Cuyvers E, Engelborghs S, Sieben A, De Roeck A (2016). Phenotypic characteristics of Alzheimer patients carrying an ABCA7 mutation. Neurology.

[93] May P, Pichler S, Hartl D, Bobbili DR, Mayhaus M, Spaniol C (2018). Rare ABCA7 variants in 2 German families with Alzheimer disease. Neurol Genet.

[94] Nuytemans K, Maldonado L, Ali A, John-Williams K, Beecham GW, Martin E (2016). Overlap between Parkinson disease and Alzheimer disease in ABCA7 functional variants. Neurol Genet.

[95] Martiskainen H, Herukka SK, Stančáková A, Paananen J, Soininen H, Kuusisto J (2017). Decreased plasma β-amyloid in the Alzheimer’s disease APP A673T variant carriers. Ann Neurol.

[96] Hansson O, Zetterberg H, Vanmechelen E, Vanderstichele H, Andreasson U, Londos E (2010). Evaluation of plasma Aβ40and Aβ42as predictors of conversion to Alzheimer’s disease in patients with mild cognitive impairment. Neurobiol Aging.

[97] Lee S, Emond MJ, Bamshad MJ, Barnes KC, Rieder MJ, Nickerson DA (2012). Optimal unified approach for rare-variant association testing with application to small-sample case-control whole-exome sequencing studies. Am J Hum Genet.

[98] Jones PA (2012). Functions of DNA methylation: islands, start sites, gene bodies and beyond. Nat Rev Genet.

[99] De Jager PL, Srivastava G, Lunnon K, Burgess J, Schalkwyk LC, Yu L (2014). Alzheimer’s disease: early alterations in brain DNA methylation at ANK1, BIN1, RHBDF2 and other loci. Nat Neurosci.

[100] Yu L, Chibnik LB, Srivastava GP, Pochet N, Yang J, Xu J (2015). Association of brain DNA methylation in SORL1, ABCA7, HLA-DRB5, SLC24A4, and BIN1 With pathological diagnosis of Alzheimer disease. JAMA Neurol.

[101] Chibnik LB, Yu L, Eaton ML, Srivastava G, Schneider JA, Kellis M (2015). Alzheimer’s loci: epigenetic associations and interaction with genetic factors. Ann Clin Transl Neurol.

[102] Reddington JP, Perricone SM, Nestor CE, Reichmann J, Youngson NA, Suzuki M (2013). Redistribution of H3K27me3 upon DNA hypomethylation results in de-repression of Polycomb target genes. Genome Biol.

[103] Humphries C, Kohli MA, Whitehead P, Mash DC, Pericak-Vance MA, Gilbert J (2015). Alzheimer disease (AD) specific transcription, DNA methylation and splicing in twenty AD associated loci. Mol Cell Neurosci.

[104] Lunnon K, Smith R, Hannon E, De Jager PL, Srivastava G, Volta M (2014). Methylomic profiling implicates cortical deregulation of ANK1 in Alzheimer’s disease. Nat Neurosci.

[105] Yamazaki K, Yoshino Y, Mori T, Yoshida T, Ozaki Y, Sao T (2017). Gene expression and methylation analysis of ABCA7 in patients with Alzheimer’s disease. J Alzheimers Dis.

[106] Ikeda Y, Abe-Dohmae S, Munehira Y, Aoki R, Kawamoto S, Furuya A (2003). Posttranscriptional regulation of human ABCA7 and its function for the apoA-I-dependent lipid release. Biochem Biophys Res Commun.

[107] Frankish A, Diekhans M, Ferreira A-M, Johnson R, Jungreis I, Loveland J (2018). GENCODE reference annotation for the human and mouse genomes. Nucleic Acids Res.

[108] Raj T, Li YI, Wong G, Humphrey J, Wang M, Ramdhani S (2018). Integrative transcriptome analyses of the aging brain implicate altered splicing in Alzheimer’s disease susceptibility. Nat Genet.

[109] Li YI, Knowles DA, Humphrey J, Barbeira AN, Dickinson SP, Im HK (2018). Annotation-free quantification of RNA splicing using LeafCutter. Nat Genet.

[110] Elsnerova K, Bartakova A, Tihlarik J, Bouda J, Rob L, Skapa P (2017). Gene expression profiling reveals novel candidate markers of ovarian carcinoma intraperitoneal metastasis. J Cancer.

[111] Liu X, Li Q, Zhou J, Zhang S (2018). ATP-binding cassette transporter A7 accelerates epithelial-to-mesenchymal transition in ovarian cancer cells by upregulating the transforming growth factor-β signaling pathway. Oncol Lett.

[112] Mohelnikova-Duchonova B, Brynychova V, Oliverius M, Honsova E, Kala Z, Muckova K (2013). Differences in transcript levels of ABC transporters between pancreatic adenocarcinoma and nonneoplastic tissues. Pancreas.

[113] Heimerl S, Bosserhoff AK, Langmann T, Ecker J, Schmitz G (2007). Mapping ATP-binding cassette transporter gene expression profiles in melanocytes and melanoma cells. Melanoma Res.

[114] Tabarés-Seisdedos R, Rubenstein JL (2013). Inverse cancer comorbidity: a serendipitous opportunity to gain insight into CNS disorders. Nat Rev Neurosci.

[115] Ibáñez K, Boullosa C, Tabarés-Seisdedos R, Baudot A, Valencia A (2014). Molecular evidence for the inverse comorbidity between central nervous system disorders and cancers detected by transcriptomic meta-analyses. PLoS Genet.

[116] Feng YCA, Cho K, Lindström S, Kraft P, Cormack J, Blalock K (2017). Investigating the genetic relationship between Alzheimer’s disease and cancer using GWAS summary statistics. Hum Genet.

[117] Witte JS, Visscher PM, Wray NR (2014). The contribution of genetic variants to disease depends on the ruler. Nat Publ Gr.

[118] Patel H, Dobson RJB, Newhouse SJ (2019) Meta-analysis of Alzheimer’s disease brain transcriptomic data. bioRxiv. 10.1101/480459 10.3233/JAD-181085 PMC6484273 30909231

[119] Grindberg RV, Yee-Greenbaum JL, McConnell MJ, Novotny M, O’Shaughnessy AL, Lambert GM (2013). RNA-sequencing from single nuclei. Proc Natl Acad Sci.

[120] Lein E, Borm LE, Linnarsson S (2017). The promise of spatial transcriptomics for neuroscience in the era of molecular cell typing. Science.

[121] Garalde DR, Snell EA, Jachimowicz D, Sipos B, Lloyd JH, Bruce M (2018). Highly parallel direct RNA sequencing on an array of nanopores. Nat Methods.

[122] Rhoads A, Au KF (2015). PacBio sequencing and its applications. Genom, Proteom Bioinforma.

[123] Jones VC, Atkinson-Dell R, Verkhratsky A, Mohamet L (2017). Aberrant iPSC-derived human astrocytes in Alzheimer’s disease. Cell Death Dis.

[124] Kelava I, Lancaster MA (2016). Dishing out mini-brains: current progress and future prospects in brain organoid research. Dev Biol.

[125] Qian H, Zhao X, Cao P, Lei J, Yan N, Gong X (2017). Structure of the human lipid exporter ABCA1. Cell.

[126] Watson M, Warr A (2019). Errors in long-read assemblies can critically affect protein prediction. Nat Biotechnol.

